# Influence of the mRNA initial region on protein production: a case study using recombinant detoxified pneumolysin as a model

**DOI:** 10.3389/fbioe.2023.1304965

**Published:** 2024-01-08

**Authors:** Filipe Fusco, Manuella Cazelato Pires, Alexandre Paulo Yague Lopes, Vítor dos Santos Alves, Viviane Maimoni Gonçalves

**Affiliations:** ^1^ Laboratory of Vaccine Development, Butantan Institute, Sao Paulo, Brazil; ^2^ Interunits Graduate Program in Biotechnology, University of Sao Paulo, Sao Paulo, Brazil

**Keywords:** recombinant *Escherichia coli*, PdT, mRNA secondary structure, translation initiation, *in silico* analysis, His-tag, bioreactor, *Streptococcus pneumoniae*

## Abstract

Recombinant proteins are of great importance in modern society, mostly as biopharmaceutical products. However, challenging and complex processes with low production yield are major drawbacks. Normally, the optimization to overcome these obstacles is focused on bioreactor and purification processes, and the biomolecular aspects are neglected, seen as less important. In this work, we present how the 5′ mRNA secondary structure region can be relevant for translation and, therefore, protein production. For this, *Escherichia coli* BL21(DE3) clones, producing recombinant detoxified pneumolysin (PdT) with and without the N-terminal His-tag, were cultivated in 10-L bioreactors. Another version of the *pdt* gene (version 2) with synonymous changes in the 5′-end nucleotide sequence was also obtained. Protein production, plasmid stability, carbon sources, and acetic acid were quantified during the cultures. Furthermore, *in silico* mRNA analyses were performed using TIsigner and RNAfold. The results showed that the His-tag presence at the N-terminus generated a minimum 1.5-fold increase in target protein synthesis, which was explained by the *in silico* mRNA analyses that returned an mRNA secondary structure easier to translate and, therefore, higher protein production than without the His-tag. The *pdt* gene version 2 showed lower 5′ mRNA opening energy than version 1, allowing higher PdT production even without a tag. This work reveals that simple mRNA analyses during heterologous gene design and production steps can help reach high-recombinant protein titers in a shorter time than using only traditional bioprocess optimization strategies.

## 1 Introduction

The global market related to recombinant proteins spent USD 1.74 billion in only 2021, with a projected increase in the following years (Polaris market Research, 2022). The practical application of these proteins varies, including pharmaceutical and biotechnology companies, academic and research institutes, and laboratory diagnostics (GVR, 2022). Although general knowledge points out the application of recombinant proteins in medicine and healthcare, their applicability is closer to our daily lives and our homes than we may think, in paper fabrication, detergents, cosmetics, textile, and food (Kapoor et al., 2017). Therefore, the production and purification processes of recombinant proteins, and their optimization, are always of great interest for producers and society.

The production of recombinant proteins is mostly performed by the cultivation of living microorganisms or mammalian cells. Another method recently developed is the *in vitro* cell-free production, which depends on the supplementation of cell components for protein synthesis (Khambhati et al., 2019). Although *in vitro* technology for recombinant protein production has progressed (Guzman-Chavez et al., 2022), the high costs of reagents and equipment availability (Kim et al., 2007) make it unfeasible for the current scale of the market necessity.

The success of the upstream process for recombinant protein production using living microorganisms is dictated by numerous factors, such as the producer organism, culture medium formulation, cultivation conditions, and characteristics of the plasmid and the heterologous gene (or insert) design (Brondyk, 2009; Sivashanmugam et al., 2009; Gustafsson et al., 2012; Barrero et al., 2021). In addition, the consecutive downstream process involves complex procedures that can limit the yield of the final product, especially if high purity is required (Vazquez et al., 2011; Soderberg, 2014; Bedade and Pawar, 2023), as is the case of pharmaceutical products. Upstream and downstream processes are intimately related, and the higher the protein concentration produced, the easier the purification process.

One of the specific challenges faced during protein synthesis is the level of mRNA translation. The mRNA can present different translational rates depending on the genetic sequence and secondary structure (Hoernes et al., 2016), consequently influencing the final amount of product recovered. For recombinant genes, the initial portion of the mRNA at the 5′-end can be modified, and the translational rate can be evaluated (Ma et al., 2002) because the site of the initiation of translation will dictate the ribosome capacity of interaction with the mRNA (Vellanoweth and Rabinowitz, 1992). This site also determines the energy required for translation initiation since the mRNA secondary structure can present loops, knots, bulges, helices, and stems, which should be disrupted for the appropriate interaction with ribosomes (Wolfsheimer and Hartmann, 2010; Achar and Saetrom, 2015).

As a result of the factors mentioned above, the insertion of N-terminal tags to promote protein solubility or facilitate protein purification modifies the 5′-end of the recombinant gene sequence and affect the translation rate (Chudakov and Lukyanov, 2003; Malhotra, 2009). For example, the addition of CAU or CAC histidine codons that generate the His-tag to facilitate the isolation of the target protein alters the mRNA structure.

Pneumolysin is a cholesterol-dependent toxin secreted by the bacterium *Streptococcus pneumoniae*. This microorganism is a leading cause of ill health and death worldwide, responsible for pneumococcal diseases such as pneumonia, meningitis, and sepsis (Roth et al., 2018; Weiser et al., 2018). Pneumolysin and its derivatives are targets for the development of new vaccines (Pichichero, 2017) and have long been produced and purified from *Streptococcus pneumoniae* and as recombinant proteins from *Escherichia coli* (Mitchell et al., 1989; Paton et al., 1991; Morgan et al., 1997; Douce et al., 2010; Marshall et al., 2015; van Pee et al., 2017; Lee et al., 2018; Vorabyev et al., 2023). However, most publications are focused on the structural or immunological aspects of pneumolysin, rather than its process development. The only work that mentions the process development reported the experimental design conducted in shaken flasks (Marini et al., 2014). Genetically detoxified pneumolysin variants are candidates for new serotype-independent pneumococcal vaccines; thus, it is essential to develop scalable and robust processes that allow high titers of protein production to make it viable for future vaccine production.

This work evaluated the production of a genetically detoxified pneumolysin, which was detoxified by three-point mutations at domain four, called recombinant PdT, to be employed as a vaccine (Berry et al., 1995). Initially, a gene construct containing the *pdt* gene without any tag was inserted into a vector for production in *E. coli*. Then, another gene construct was prepared by adding the codons of the His-tag at the 5′-end of the target gene, and the gene sequence for tobacco etch virus (TEV) protease recognition for posterior tag removal (Raran-Kurussi et al., 2017), before the *pdt* gene sequence. The PdT and His–TEV–PdT products were obtained in a 10-L bioreactor, and their production levels were evaluated. Due to the great difference in the production levels of both recombinant proteins, we evaluated the effect of the 5′ region of mRNA on protein synthesis by *in silico* analysis focused on the mRNA structure and its impact on heterologous gene expression. Finally, the *in silico* analysis and protein production data of a new *pdt* gene construct with the modified mRNA initial region helped clarify the differences observed and, therefore, highlight the importance of the 5′-end genetic region for recombinant protein production.

## 2 Materials and methods

### 2.1 Gene construct and cell bank preparation

The codons of the *pdt* gene (Berry et al., 1995) were optimized for *E. coli* codon usage and synthesized by GenOne Biotechnologies™ (Rio de Janeiro, Brazil), which supplied it into the pET28a+ plasmid (Novagen/Merck, Darmstadt, Germany). This construct (*pdt* gene version 1) was used as a template for the insertion of codons of the N-terminal His-tag and the sequence of the TEV protease cleavage site. A *Bam* H1 restriction site was included between the codons of glycine and serine found inside the sequence of the TEV protease cleavage site, allowing us to remove the additional 5′-end sequences and recover the optimized *pdt* gene sequence, if necessary. Two steps of the polymerase chain reaction (PCR) using designed primers (Thermo Fisher™, Waltham, Massachusetts, EUA) were performed. First, the gene sequence of the TEV cleavage site was inserted using primers 1F and 3R (Table 1). Second, the His-tag gene sequence was inserted using primers 2F and 3R (Table 1). After each PCR, 1% agarose gel electrophoresis was run to confirm insertion (Sambrook and Russel, 2001), and the final PCR product was purified using a commercial kit (QIAquick, QIAGEN™, Hilden, Germany).

**TABLE 1:** Primers used for *his-tev-pdt* insert construction and gene sequencing. F: forward; R: reverse; and bp: base pairs.

Primer	Sequence	Region
1F	5’gaa​aac​ctg​tat​ttc​cag​gga​tcc​gcc​aat​aaa​gcc​gtg​aac’3	29–71 bp
2F	5’cga​cca​tgg​gtc​acc​acc​atc​atc​atc​acg​aaa​acc​tgt​att​tcc​agg​g’3	1–49 bp
3R	5’cag​tct​cga​gtt​agt​cgt​ttt​cca​ctt​tat​ctt​cc’3	1,441–1,476 bp
4F	5’gaa​aca​gcg​tgg​tat​tag​cg’3	719–1,476 bp
5R	5’ctg​gtg​gtt​tcc​agt​ttc​ag’3	812–1 bp

The purified amplicon (*his-tev-pdt* gene) was quantified using the NanoDrop Spectrophotometer 2000 (Thermo Fisher™, Waltham, Massachusetts, EUA). The amplicon and the pET28a+ plasmid were digested with *Nco* I and *Xho* I restriction enzymes (New England Biolabs™, Ipswich, Massachusetts, EUA), purified, and ligated using T4 ligase (New England Biolabs™, Ipswich, Massachusetts, EUA) at 16°C for 18 h. After ligation, the pET28a+*his-tev-pdt* gene product was used to transform *E. coli* DH5α (Invitrogen™, Waltham, Massachusetts, EUA). *E. coli* DH5α was also transformed with the pET28a+*pdt* plasmid. The *E. coli* DH5α transformants were cultivated in lysogeny broth with agar (LB-agar) and 50 mg/L kanamycin (Kan) for 24 h at 37°C. To confirm transformation, isolated colonies were subjected to PCR using primers 2F and 3R (Table 1). The plasmids of the PCR-positive colonies were purified (QIAprep Spin Miniprep Kit, QIAGEN, Hilden, Germany) and selected for sequencing (Sanger et al., 1977) (3500 Genetic Analyzer Hitachi High Technologies™, Tokyo, Japan) using primers 4F and 5R (Table 1) and T7 forward (F) and reverse (R) promoter primers. One clone presenting the correct sequence was used to prepare the cell bank in the LB–Kan medium with 15% glycerol.

The plasmid DNA was purified (kit 27.104, QIAGEN, Hilden, Germany) from *E. coli* DH5α and employed to transform competent *E. coli* BL21(DE3) cells (Thermo Fisher™, Waltham, Massachusetts, EUA) using the same procedures described for *E. coli* DH5α transformation. To evaluate gene expression, five colonies isolated from M9 minimal medium (M9)–agar–Kan were transferred to the liquid M9–Kan medium and cultivated at 37°C and 250 rpm. When the optical density at 600 nm (OD_600_) reached 0.8, the cultures were induced with 0.5 mM isopropyl β-D-1-thiogalactopyranoside (IPTG) for 3 h at 30°C. Samples were collected before and after induction to measure OD_600_ and quantify target proteins. Clones that showed higher levels of target protein production were chosen to prepare master and working cell banks in the M9–Kan medium containing 15% glycerol. The vials of the cell banks were filled with a cell suspension sufficient to yield OD_600_ = 0.1 when inoculated into 100 mL of medium, and the vials were stored at −80°C until further use.

### 2.2 Inoculum preparation

The inoculum was prepared at 37°C and 300 rpm for approximately 3 h by the addition of two vials of the working cell bank into two 300-mL Tunair^TM^ flasks (one vial per flask), with each flask containing 100 mL of medium (Table 2).

**TABLE 2:** Culture medium composition for inoculum preparation.

Component	Final concentration	Unit
Glycerol	10.0	g/L
Glucose	1.0	
Yeast extract	5.0	
Peptone	10.0	
KH_2_PO_4_	3.4	
Na_2_HPO_4_	9.0	
NH_4_Cl	2.7	
Na_2_SO_4_	0.7	
MgSO_4_∙7H_2_O	0.5	
EDTA	14.1	mg/L
CoCl_2_∙6H_2_O	2.5	
MnCl_2_∙4H_2_O	15.0	
CuCl_2_∙2H_2_O	1.5	
H_3_BO_3_	3.0	
Na_2_MoO_4_∙2H_2_O	2.1	
Zn(CH_3_COO)_2_∙2H_2_O	33.8	
Iron (III) citrate	100.8	
Kanamycin sulfate	50.0	
Thiamine	45.0	
Polypropylene glycol	0.03	% v/v

### 2.3 PdT and His–TEV–PdT production in the bioreactor

The bioreactor cultures were carried out using an auto-induction medium, which contains 10 g/L glucose, 20 g/L lactose, and 30–40 g/L glycerol in the same medium composition given in Table 2. A specific volume of the inoculum was transferred to the 10-L bioreactor (Biostat Cplus, Sartorius™, Gottingen, Germany), with 6–7 L of the auto-induction medium, to reach an initial OD_600_ between 0.1 and 0.2. During the culture, dissolved oxygen was set in 30% of saturation and controlled by a cascade of air flow (1–7 L/min), impeller speed (150–1,000 rpm), and pure oxygen injection (0%–100%). pH was maintained at 7.0 by NH_4_OH 25% (v/v) addition. The temperature was maintained initially at 37°C and later at 25°C. The cultivation finished when carbon sources were exhausted, as indicated by the abrupt increase in the dissolved oxygen level.

Two different temperature conditions were evaluated in this work for both proteins. For condition 1 (C1), the temperature was changed from 37°C to 25°C after 4 h of exponential growth. For condition 2 (C2), the temperature was changed from 37°C to 25°C after glucose exhaustion.

During the cultivation process, samples were collected at 1- or 2-h intervals to measure OD_600_, separate the pellet and supernatant after centrifugation, quantify the cell dry mass (CDM), and analyze plasmid stability. After cultivation, the cells were harvested by centrifugation and frozen at −20°C for further use.

### 2.4 Target protein analyses

During each culture, a volume of the cell suspension was collected to obtain pellets that present OD_600_ = 5.0 when resuspended in 1 mL of disruption buffer. The disruption buffer was composed of BugBuster 10X (EMD Millipore, Billerica, Massachusetts, United States), 20 mM bis-tris (Sigma-Aldrich, St. Louis, Missouri, United States), Benzonase nuclease (EMD Millipore, Burlington, Massachusetts, United States), an EDTA-free protease inhibitor cocktail (Roche Diagnostics GmbH, Mannheim, Germany), and lysozymes (Sigma, St. Louis, Missouri, United States), as recommended by the BugBuster user guide. After resuspension, the biomass was incubated in a shaking platform at room temperature for 20 min. The lysate was centrifuged for 20 min at 16,000 g, the supernatant (soluble fraction) was separated from the pellet (insoluble fraction), and the pellet was resuspended in 3 M urea to the original volume of 1 mL.

The target proteins were quantified in the supernatant and pellet samples. The total protein concentration was measured by bicinchoninic acid assay (Smith et al., 1985) using a BCA kit (Thermo Fisher Scientific, Rockford, Illinois, United States). Then, sodium dodecyl sulfate-polyacrylamide gel electrophoresis (SDS-PAGE) was performed. The gels were stained with Coomassie blue R-250 (Life Technologies, Grand Island, New York, United States) and scanned using a densitometer DS-5000 with L-Pix Image v.2.11.7 software (Loccus, Sao Paulo, Brazil). The densitometry of gel bands was performed using LabImage v.4 software (Kapelan Bio-Imaging, Leipzig, Germany) to determine the relative quantity of target proteins in each lane (Eq. 1). The target protein concentration was estimated according to Eq. 2.

$$RQ\left(\%\right)=\frac{TP\;band\;intensity\;}{\sum intensity\;of\;all\;bands\;in\;the\;lane}\times 100,$$ (1)

where RQ is the relative quantity (%) of the target protein (TP).

$$\left[TP\right]=\frac{total\;protein\;concentration\times RQ}{100},$$ (2)

where [TP] is the target protein concentration and RQ is the relative quantity.

### 2.5 Supernatant and cell dry mass analyses

The culture supernatant samples were analyzed by high-performance liquid chromatography (HPLC) (1260 Infinity, Agilent™, Santa Clara, California, EUA) for carbon source and acetic acid quantification. An Aminex HPX-87H column (Bio-Rad Laboratories™, Hercules, California, EUA) was used, with 5 mM H_2_SO_4_ as the mobile phase and a flow rate of 0.6 mL/min at 60°C. Carbon sources were detected using the refraction index and acetic acid by UV at 210 nm.

The CDM quantification was based on the weight of 0.22-µm membranes of mixed cellulose esters (Millipore™, Burlington, Massachusetts, United States). The membranes were previously dried at 60°C until constant weight was obtained, weighted as the blank, used to filter a specific volume of the culture, in duplicate, and weighted after the content dried the same way as described above. The values obtained were used to determine a correlation between OD_600_ and cell dry mass.

To measure the plasmid stability, 100 µL of serial dilutions of each bioreactor sample (10^6^ or 10^7^) were plated on LB–agar plates, and the plates were incubated overnight at 37°C. Then, 70 colonies were replicated in two new plates: LB–agar–Kan and LB–agar. The plates were incubated under the same conditions as before. The plasmid stability was estimated by the quotient of colonies that grew with and without the antibiotic.

### 2.6 *In silico* analyses

The *pdt* and *his-tev-pdt* gene sequences were submitted for software translation initiation coding region designer (https://tisigner.com/) analysis with a default configuration to determine the mRNA opening energy and expression score. TIsigner analyzes the mRNA translation initiation region, which is one of the most impacting factors in gene expression. This software application can also suggest synonym mutations of the first nine codons to minimize the opening energy and, therefore, enhance gene expression, but this function was not used in this work (Bhandari et al., 2021a).

The region from the nucleotide positions −30 to +30 (−30:30) of each gene construct was submitted to RNAfold from the ViennaRNA Package version 2.5.1 (http://rna.tbi.univie.ac.at/cgi-bin/RNAWebSuite/RNAfold.cgi) (Gruber et al., 2008) analysis with a default configuration for mRNA secondary structure analysis. RNAfold is a software application that can predict the RNA secondary structure based on the minimum free energy (MFE), making it possible to evaluate structures that are more stable and, therefore, more difficult to be opened and translated.

Finally, to verify the estimated half-life of both proteins and evaluate its impact on the target protein amount, the amino acid sequences were submitted to the ProtParam tool at Expasy (https://web.expasy.org/protparam/) (Gasteiger et al., 2005).

### 2.7 Modification of the PdT mRNA initial region

To clarify the impact of the mRNA initial region on PdT production, another version of the *pdt* gene (version 2) was inserted into the pET28a+ vector between *Xho* I and *Nco* I and transformed into *E. coli* BL21(DE3) cells, as described in the previous sections. This gene sequence was not submitted for codon usage optimization for *E. coli*; i.e., it is the original sequence (Gene ID: 66806991) with three mutations, C428G, W433F, and D385N, as described by Berry et al. (1995). The first codons of this sequence are distinct from those of the *pdt* gene version 1 (Supplementary Figure S1) and were analyzed using the same bioinformatics tools already mentioned. Moreover, this strain was cultivated under C1 also following the same methodology described before.

## 3 Results

### 3.1 Genetic insert construction

The optimized *pdt* gene sequence presented 78.8% similarity with the original *pdt* sequence. After PCRs were conducted for the addition of the gene sequences of the TEV protease cleavage site and His-tag, the number of amino acids and nucleotides increased from 472 and 1,416 to 487 and 1,461, respectively. The genetic sequences are shown in Supplementary Table S1, and the representation of the gene constructs is shown in Supplementary Figure S1. In the *his-tev-pdt* sequence, the start codon of the untagged *pdt* sequence was removed, and a new ATG start codon was included before the *his-tag*-encoding sequence (Supplementary Table S1).

### 3.2 Bioreactor cultures

The cultures comparing PdT and His–TEV–PdT production were performed under two different conditions, C1 and C2. In C1, the temperature was reduced from 37°C to 25°C after 4 h of the beginning of the exponential growth phase, while in C2, the temperature was shifted when glucose was exhausted. The most relevant results are shown in Table 3.

**TABLE 3:** Summary of the most important results of 10-L bioreactor cultures for PdT and His–TEV–PdT production. C1—temperature was reduced from 37°C to 25°C 4 h after the beginning of the exponential growth phase. C2—temperature was shifted from 37°C to 25°C when glucose was exhausted. μ_max_—maximum specific growth rate. Acetic_max_—maximum acetic acid concentration. CDM_max_—maximum cell dry mass. Soluble_max_—maximum target protein obtained in the soluble fraction.

Protein (Condition)	µ_max_ (h^−1^)	Acetic_max_ (g/L)	CDM_max_ (g/L)	Soluble_max_ (g/L)
PdT (C1)	1.08	1.1	23.6	1.7
PdT (C2)	0.94	1.7	25.4	1.8
His-TEV-PdT (C1)	0.72	2.0	32.0	2.5
His-TEV-PdT (C2)	0.78	1.8	36.8	3.2

**FIGURE 1:**
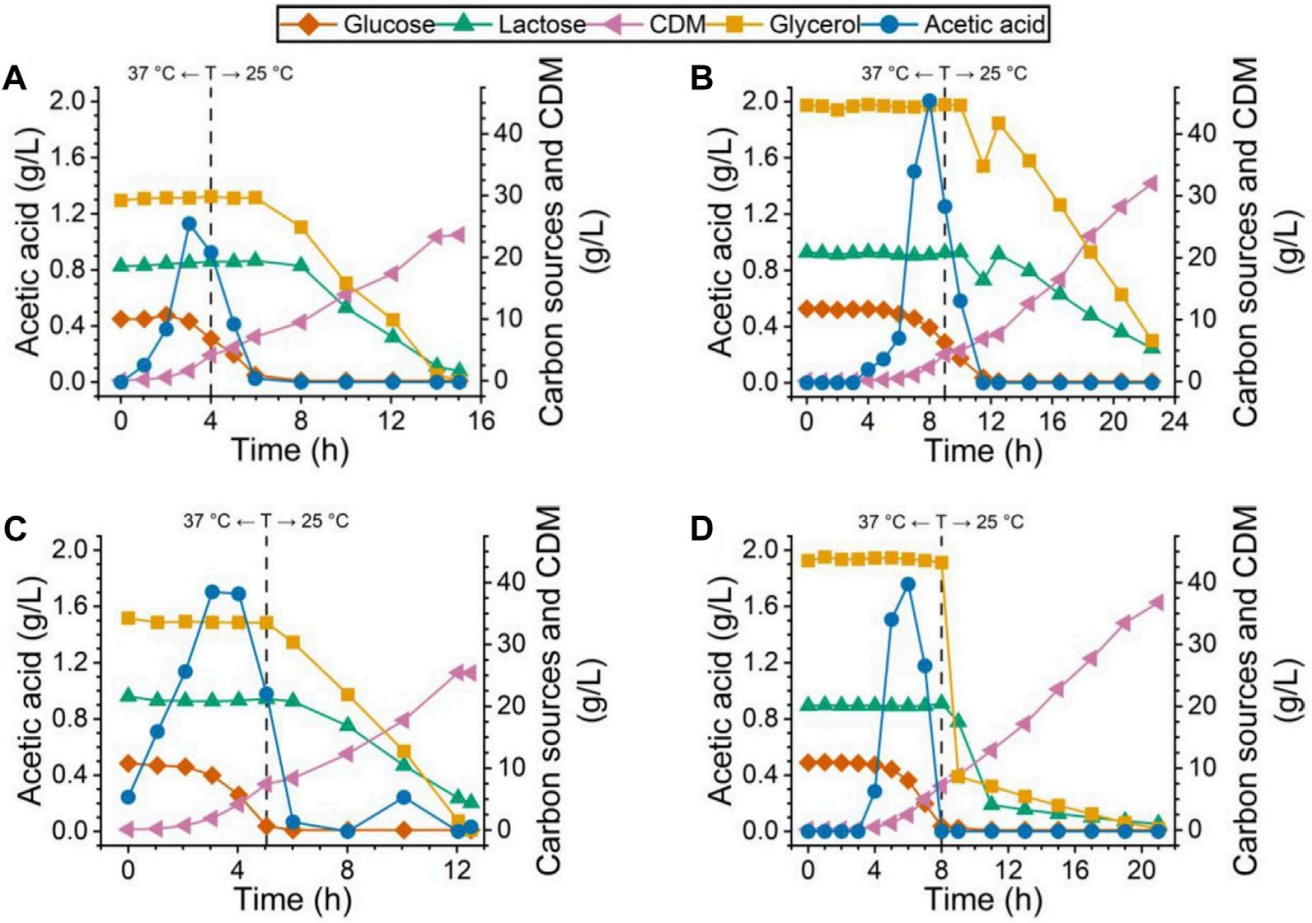
Carbon source, acetic acid, and CDM profile for all four bioreactor cultures. Cultures for **(A)** PdT and **(B)** His–TEV–PdT production under C1 (temperature reduction 4 h after the beginning of the exponential growth phase). Cultures for **(C)** PdT and **(D)** His–TEV–PdT production under C2 (temperature reduction when glucose was exhausted). Dashed lines indicate the moment when the temperature was reduced from 37°C to 25°C.

The maximum specific growth rate (µ_max_) was 1.08 h^-1^ for PdT production and 0.72 h^-1^ for His–TEV–PdT production under C1. Under C2, µ_max_ was 0.94 h^-1^ for PdT production and 0.78 h^-1^ for His–TEV–PdT production (Table 3). These results indicate that PdT-producer cells grew faster than His–TEV–PdT producers. It is noteworthy that the cultures presented a maximum acetic acid production ≤2.0 g/L (Table 3). After reaching the maximum values, the acetic acid concentration started to decrease when glucose concentration was lower than 8.0 g/L (Figures 1A–D).

Concerning the biomass, cultures producing His–TEV–PdT protein (Figures 1B, D), regardless of the condition, started with a slightly higher glycerol concentration and reached higher values of CDM than cultures for PdT production (Figures 1A, C). Thus, the substrate-to-cell conversion factor (Yx/s) was calculated for glycerol, which returned 0.54 g cells/g glycerol for the PdT production cultures against 0.67 g cells/g glycerol for the His–TEV–PdT producers. Except to the fact that cultures showed higher CDM values under C2 (Figures 1C, D), no other effects were noted due to a temperature shift at different moments. Furthermore, it can also be seen that glycerol and lactose consumption just started when glucose was completely exhausted. Then, glycerol was consumed first, while lactose was metabolized later. Finally, during His–TEV–PdT production under C2 (Figure 1D), glycerol and lactose concentrations showed a very steep decrease, while it was not observed in the other cultures.

### 3.3 Target protein analyses

**FIGURE 2:**
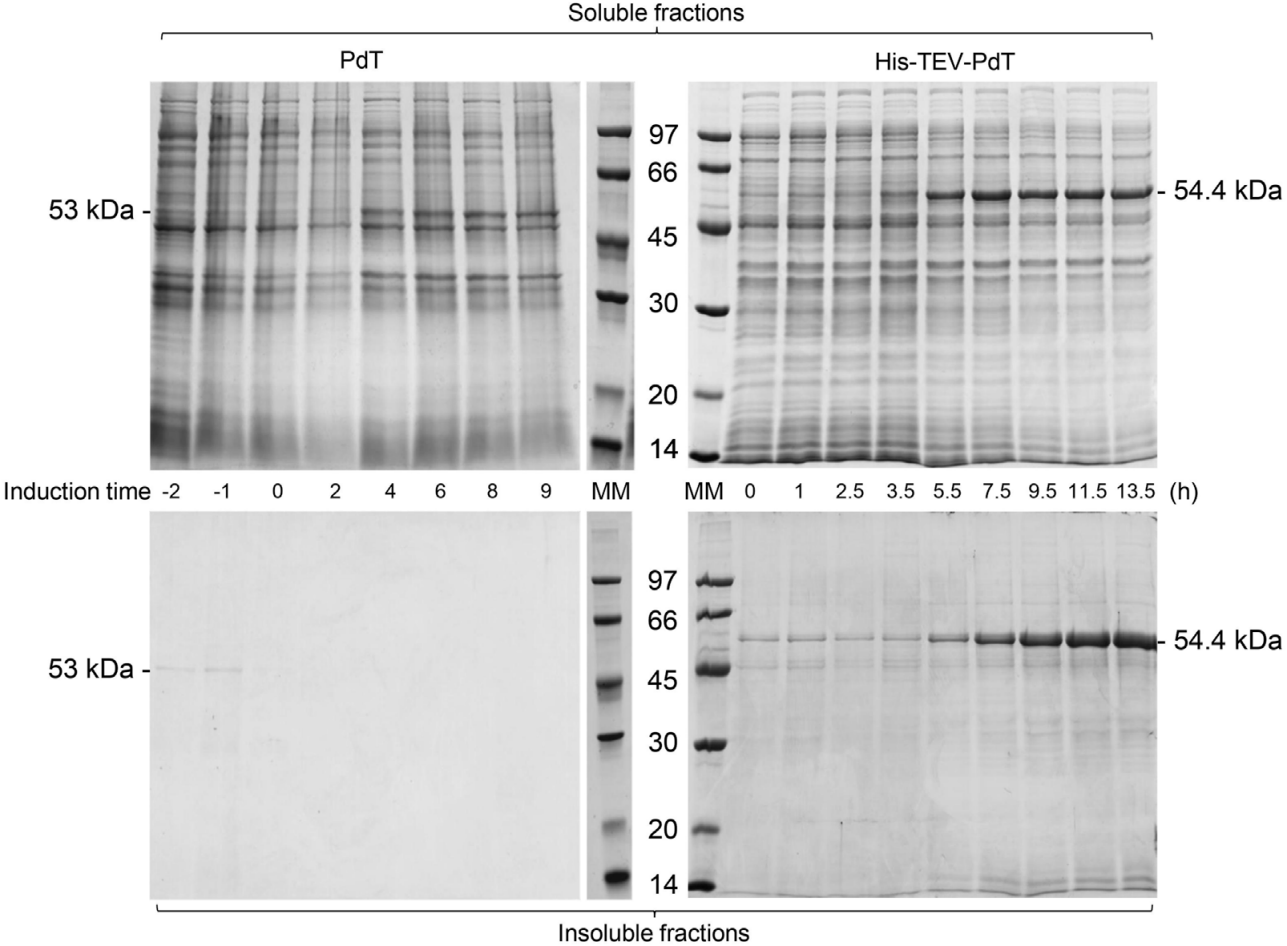
SDS-PAGE of the supernatant (soluble fractions) and pellets (insoluble fractions) of samples of *E. coli* BL21(DE3) cell cultivation to produce PdT (53 kDa) or His–TEV–PdT (54.4 kDa) under C1 (temperature reduction 4 h after the beginning of the exponential growth phase). Culture samples were lysed with BugBuster, and a protein amount corresponding to OD_600_ of 5.0 was applied per lane. Electrophoresis with 12% gels was conducted under reducing conditions. MM: molecular marker.

**FIGURE 3:**
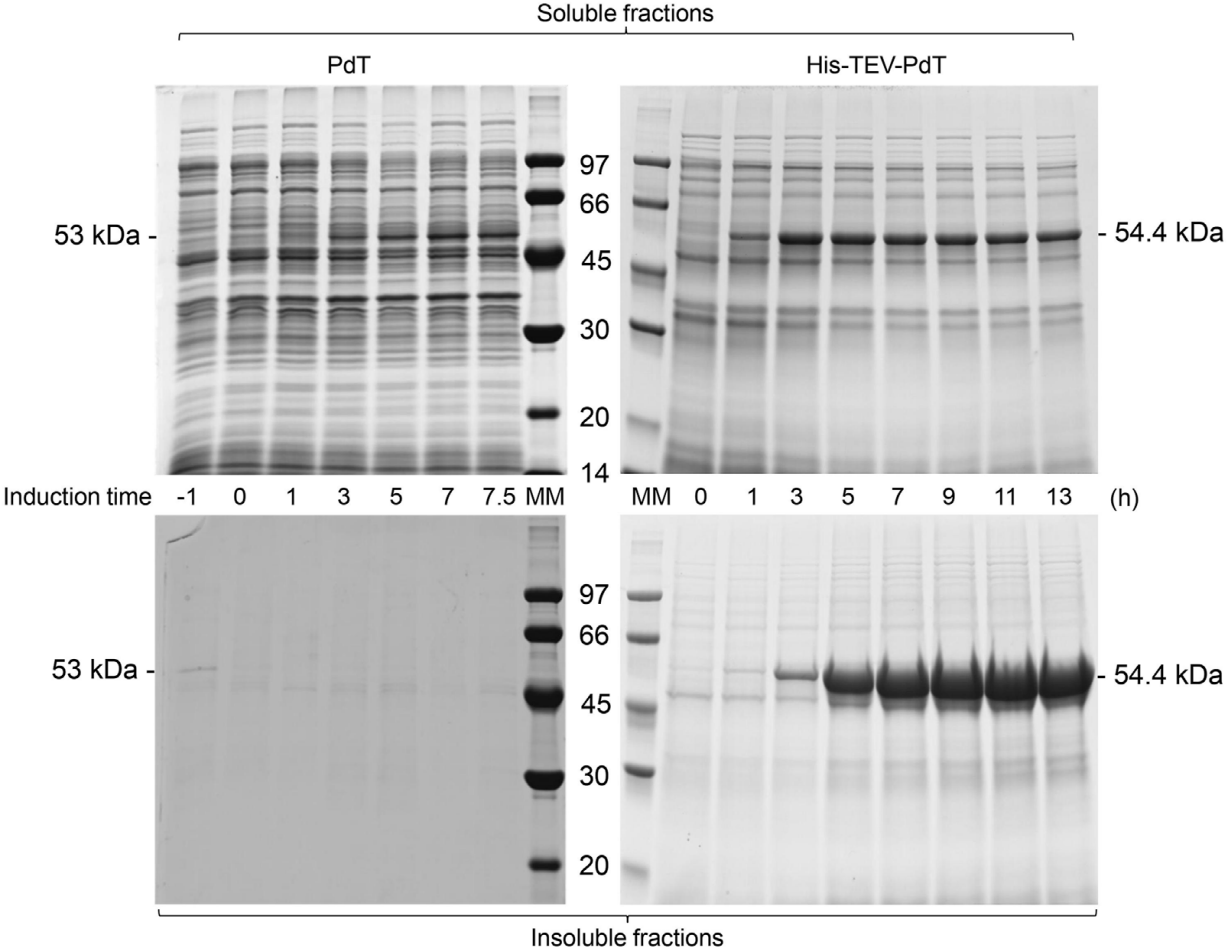
SDS-PAGE of the supernatant (soluble fractions) and pellets (insoluble fractions) of samples of *E. coli* BL21(DE3) cell cultivation to produce PdT (53 kDa) or His–TEV–PdT (54.4 kDa) under C2 (temperature reduction when glucose was exhausted). Culture samples were lysed with BugBuster, and a protein amount corresponding to OD_600_ of 5.0 was applied per lane. Electrophoresis with 12% gels was conducted under reducing conditions. MM: molecular marker.

The proteins with and without the His-tag were produced during the *E. coli* BL21(DE3) cell cultivation (Figures 2, 3). Under C1, SDS-PAGE showed that a PdT band appeared in the soluble fraction 4 h after the beginning of lactose consumption, while His–TEV–PdT production started 3.5 h after the beginning of lactose consumption (Figure 2, upper panel).

The insoluble fractions of the culture samples were also analyzed. These fractions were composed of cellular debris and insoluble proteins that aggregated to form inclusion bodies. SDS-PAGE showed that the PdT protein was absent from insoluble fractions, but the insoluble His–TEV–PdT protein was observed after 5.5 h induction (Figure 2, bottom panel). A band of the same size as the His–TEV–PdT protein (54.4 kDa) was also observed in the first 3.5-h induction, but Western blot showed that it was not the target protein (data not shown).

Under C2, SDS-PAGE showed that the PdT band appeared in the soluble fraction 3 h after induction, while His–TEV–PdT production started after 1 h (Figure 3, upper panel). As under C1, the PdT protein did not appear in C2 insoluble fractions, but His–TEV–PdT bands were clearly observed in insoluble fractions 3 h after induction (Figure 3, bottom panel).

**FIGURE 4:**
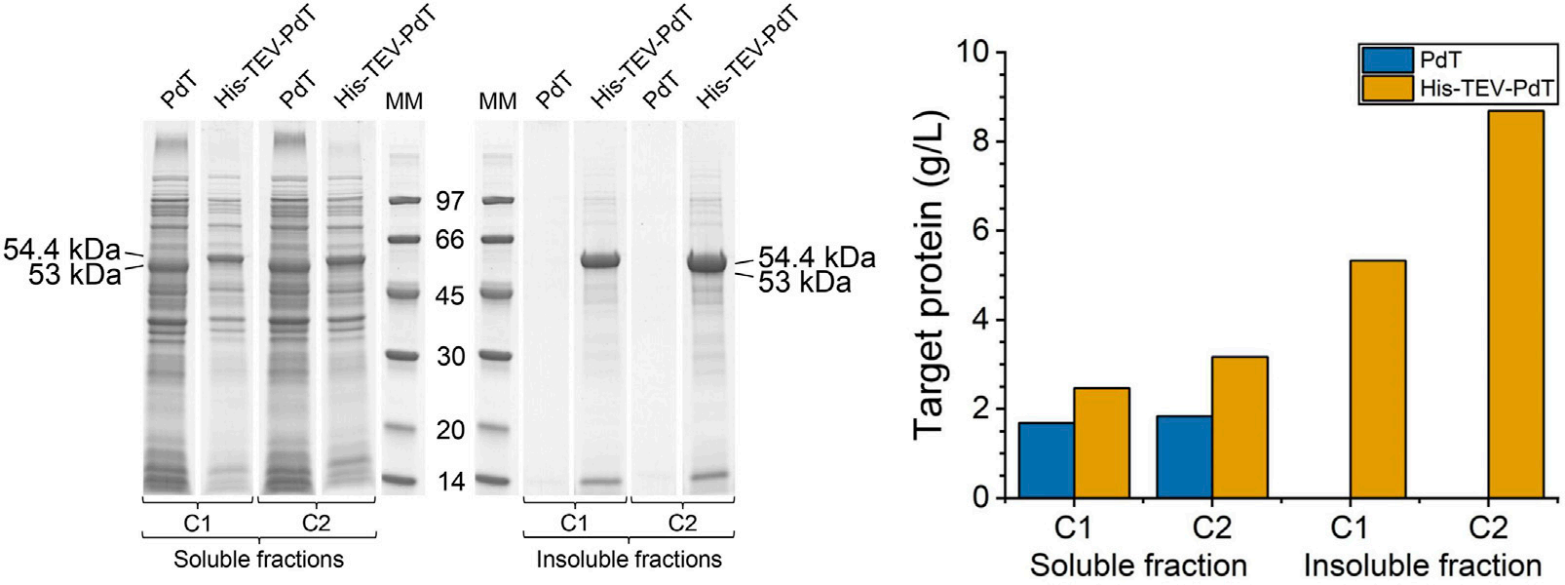
Left panel: SDS-PAGE to compare PdT and His–TEV–PdT production in the final wet biomass processed in parallel. Under cultivation C1, the temperature was reduced from 37°C to 25°C 4 h after the beginning of the exponential growth phase, and under C2, the temperature was reduced when glucose was exhausted. Culture samples were lysed with BugBuster, and a protein amount corresponding to OD_600_ of 5.0 was applied per lane. Electrophoresis with 12% gels was conducted under reducing conditions. MM: molecular marker. Right panel: Estimated concentration of PdT and His–TEV–PdT in grams per liter of culture, calculated using Eq. 2.

To evaluate whether the differences found in the amounts of PdT and His–TEV–PdT produced were not due to sample manipulation, the wet biomass of each culture was analyzed simultaneously for comparison purposes. The amount of wet biomass processed was sufficient to achieve an OD_600_ value equal to 5.0 in 1 mL of lysis buffer. The total protein concentration was measured by BCA, and the RQ of target proteins was measured by densitometry of the SDS-PAGE bands (Figure 4, left panel). The results confirmed that the amount of the His–TEV–PdT produced was higher than the amount of PdT (Figure 4, right panel). In addition, only His–TEV–PdT was found in insoluble fractions (Figure 4, left panel), and its concentration in these fractions was higher than in soluble fractions, especially under C2 (Figure 4, right panel). The comparison of PdT production showed that cultivation conditions had little, if any, impact on soluble protein titers (Figure 4, right panel). Although significant amounts of His–TEV–PdT were obtained as inclusion bodies under C2, their use in antigen purification would require extra steps to solubilize and refold the protein, which does not guarantee the correct structural conformation of the antigen and could comprise the immune response.

In terms of plasmid stability, during PdT production, >95% of cells retained the plasmid after induction under both cultivation conditions (Table 4). On the other hand, during His–TEV–PdT production, although plasmid stability was >97% before induction, an intense plasmid loss was observed after induction under both cultivation conditions (Table 4).

**TABLE 4:** Plasmid stability. BI—immediately before induction; AI—after induction (last sample collected before the culture was finished).

	Target protein and condition			
Moment	PdT	PdT	His–TEV–PdT	His–TEV–PdT
	C1	C2	C1	C2
BI	100%	98.6%	97.1%	98.6%
AI	95.7%	97.1%	0%	0%

### 3.4 *In silico* analysis

**FIGURE 5:**
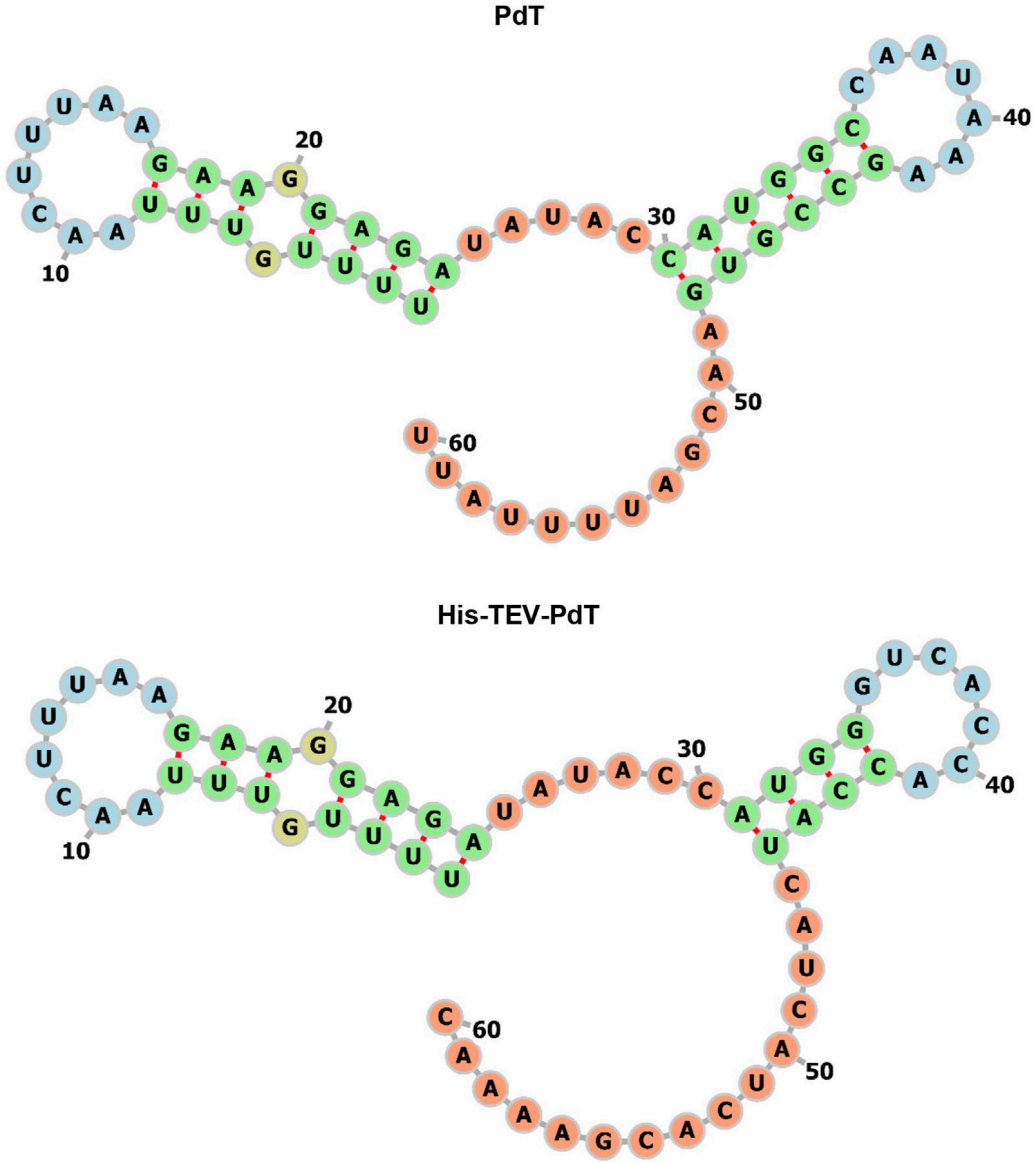
−30:30 region of the mRNA MFE secondary structure predicted by RNAfold for the translation of *pdt* and *his-tev-pdt* genes. The numbers indicate the ribonucleotide position from the beginning of the sequence used as input. Start codons are located from 31 to 33, considering the figure numbers. The colors represent the different structures observed. Green—stems, yellow—interior loops, blue—hairpin loops, and orange—5′ and 3′ unpaired regions.

To elucidate the fact that it is easier to produce His–TEV–PdT than PdT, three analyses of bioinformatics were performed. The first analysis was carried out using TIsigner with the objective to evaluate the mRNA opening energy and the expression score. The results showed that the mRNA for *pdt* gene translation has an opening energy of 14.19 kcal/mol and an expression score of 33.6, while the mRNA for *his-tev-pdt* gene translation showed 8.7 kcal/mol and 90.72, respectively. These results may explain why, when produced under the same condition, His–TEV–PdT is obtained in higher amounts than PdT. The second analysis was conducted using RNAfold with the objective of predicting the mRNA structure to find possible structural characteristics that may interfere with ribosome binding and translation initiation. The result of the analysis of the −30:30 mRNA region is shown in Figure 5, and it can be seen that the ribosome should face a longer stem structure for *pdt* translation, which may require more energy to be opened, as the TIsigner analysis revealed, and, therefore, negatively affect the translation process. Furthermore, RNAfold returned an MFE of −9.30 kcal/mol for *pdt* gene translation against −4.30 kcal/mol for *his-tev-pdt* gene translation, indicating a more stable secondary structure of mRNA encoding the *pdt* gene. The final analysis using the ProtParam tool at Expasy calculated the estimated half-life in *E. coli* of both proteins as >10 h, which is the maximum value, indicating that both proteins should be equally stable, and PdT proteolytic degradation during cultivation was probably not the reason for low titers.

### 3.5 Modification of the PdT mRNA initial region

**FIGURE 6:**
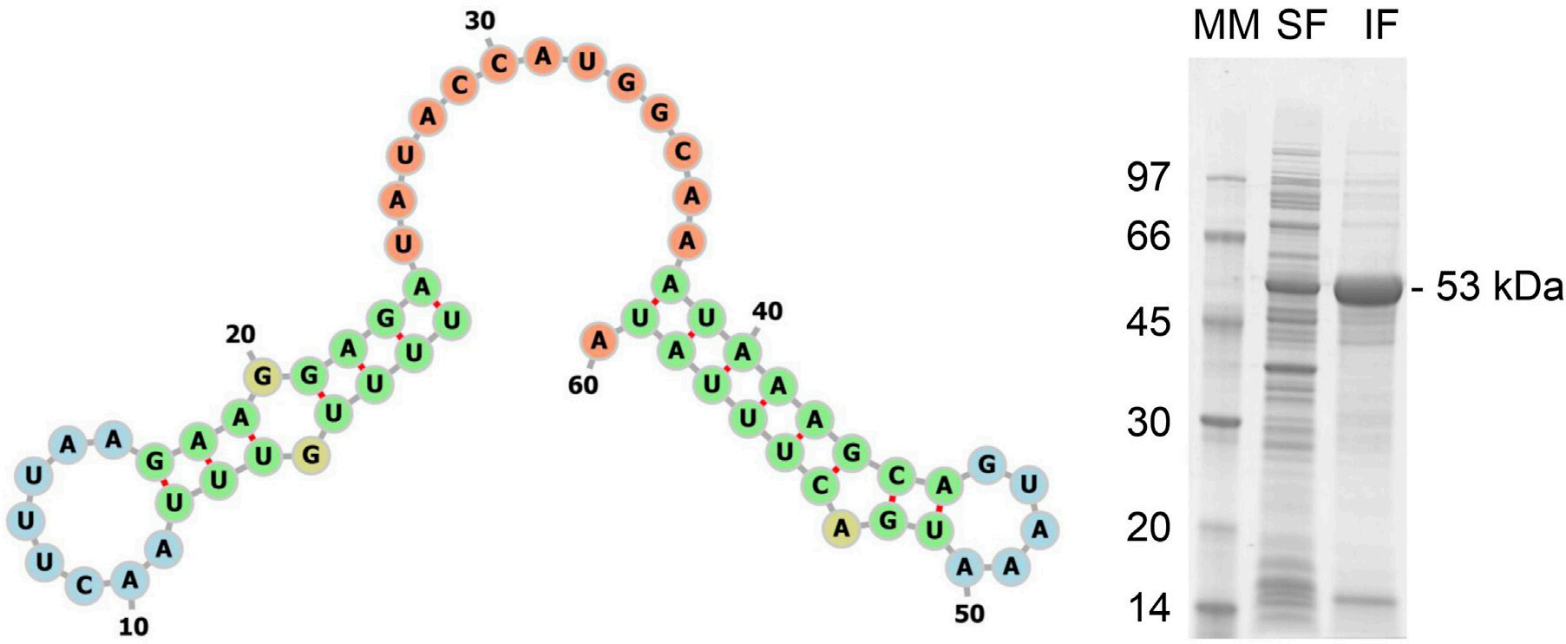
Left panel: −30:30 region of the mRNA MFE secondary structure predicted by RNAfold for the translation of the *pdt* gene version 2. The numbers indicate the ribonucleotide position from the beginning of the sequence used as input. Start codons are located from 31 to 33, considering the figure numbers. The colors represent the different structures observed. Green—stems, yellow—interior loops, blue—hairpin loops, and orange—5′ and 3′ unpaired regions. Right panel: SDS-PAGE of the supernatant (soluble fraction—SF) and pellet (insoluble fraction—IF) of the final wet biomass of *E. coli* BL21 (DE3) cultivation to produce PdT (53 kDa) from gene version 2. Samples were lysed with BugBuster, and a protein amount corresponding to OD_600_ of 5.0 was applied per lane. Electrophoresis with 12% gels was conducted under reducing conditions. MM: molecular marker.

The *pdt* genetic sequence version 2 was analyzed using TIsigner, returning an opening energy of 9.8 kcal/mol and an expression score of 83.27. The RNAfold analysis returned an MFE of −5.20 kcal/mol and the −30:30 mRNA secondary structure, as shown in Figure 6 (left panel). The ProtParam analysis, as before, returned an estimated half-life > 10 h in *E. coli*. These values are between the results obtained from the first version of *pdt* and *his-tev-pdt* gene sequences, suggesting that the modified PdT production should be between the two previous target proteins (Table 5).

**TABLE 5:** *In silico* analysis of the mRNA 5′-end influence on the translation of PdT and His–TEV–PdT.

Gene sequence	Opening energy (kcal/mol)	Expression score	MFE (kcal/mol)	Soluble_max_ under C1 (g/L)
*his-tev-pdt*	8.70	90.72	−4.30	2.5
*pdt* version 1	14.19	33.60	−9.30	1.7
*pdt* version 2	9.80	83.27	−5.20	2.0

The SDS-PAGE from the final sample of the culture performed to produce PdT from the gene sequence version 2 is shown in Figure 6 (right panel), where a clearly bigger band can be observed when compared to the PdT obtained using the gene sequence version 1. Quantification returned 2.0 g/L in soluble fraction and 7.8 g/L in insoluble fraction, which is in accordance with the *in silico* results (Table 5).

## 4 Discussion

The auto-induction medium used in this work to produce the untagged PdT and His–TEV–PdT proteins exploits the catabolite repression mechanism promoted by the glucose presence. Therefore, lactose is not consumed initially, and induction takes place only when glucose is exhausted (Görke and Stuike, 2008). Related to that, it is noteworthy that after glucose exhaustion, glycerol is consumed first and then lactose. It happens because cells need to prepare the enzymatic machinery to metabolize lactose. On the other hand, glycerol at high concentrations does not need transporters; it is internalized by passive diffusion (Voegele et al., 1993) and consumed rapidly, serving as an extra source of energy while the cells prepare for lactose consumption.

Cultures producing His–TEV–PdT reached higher CDM values compared to PdT producers because they started with a slightly higher glycerol concentration than the others, which means more energy to grow. Another reason for the differences in CDM is the higher conversion from glycerol to cells of His–TEV–PdT producers, achieving higher numbers of cells than PdT producers when consuming the same amount of glycerol. In addition, cultures under C2 reached higher CDM values, indicating that the temperature shift from 37°C to 25°C when glucose was depleted was advantageous for cell growth when compared to the temperature shift 4 h after the beginning of the exponential growth phase.

Acetic acid production and accumulation is a very important factor for *E. coli* cultivation since it can inhibit growth and recombinant protein production at different intensities, depending on the culture medium and the cell strain, whether it is a recombinant or not (Koh et al., 1992; Sun et al., 1993). Acetic acid production takes place when bacterial growth and carbon source uptake are so fast that the formation of products cannot happen at the same speed, so the carbon source excess is destined to fermentative pathways (De Mey et al., 2007). Our cultures reached values between 1.1 and 2.0 g/L, which are reported as inhibitory in some cases (Sun et al., 1993; Pinhal et al., 2019). Here, the maximum acetate concentration was observed within the interval in which cells grew at µ_max_ in each culture, which indicates that no growth inhibition happened. Thus, our cells in this medium might be tolerant to higher acetate concentrations than reported in these works. After a temperature decrease, acetic acid concentration decreased due to the reduction in the carbon source uptake rate. In addition, after glucose exhaustion, acetic acid was consumed as the cells are no longer under catabolic repression.

Regarding target protein production, C2 returned the best results for both target proteins. This is probably due to the higher CDM concentrations achieved. Comparing the two target proteins, clearly, the production of His–TEV–PdT was much more intense. In addition, the His–TEV–PdT production was always detected earlier than the PdT production. Moreover, different from the untagged PdT protein, the His–TEV–PdT protein was also found in larger amounts in the insoluble fraction than in the soluble fraction, probably due to incorrect folding promoted by strong overexpression (Beygmoradi et al., 2023). Notably, His–TEV–PdT production was detected after only 1 h of induction under C2 and after 3.5 h under C1. In addition, rapid glycerol and lactose consumption was observed after induction under C2, which corroborated with the faster His–TEV–PdT production under this condition than under C1.

To evaluate whether an increase in the initial glycerol concentration could enhance PdT production to reach a similar amount as His–TEV–PdT, these results were compared to those of another culture for PdT production that was conducted with 80 g/L of glycerol under C2 (data not shown). However, even with twice the glycerol amount, PdT production was lower than the His–TEV–PdT production with 40 g/L glycerol, supporting the hypothesis that something else should be responsible for these differences in target protein production.

Another possible cause for higher His–TEV–PdT production could be the differences in the half-life between the target proteins. However, there is no recognition site to direct both proteins to the degradation machinery in their amino acid sequences since there are no primary (leucine, phenylalanine, tyrosine, and tryptophan) or secondary (arginine and lysine) destabilizing N-terminal residues in the proteins, as shown in Supplementary Table S2. Even if considering the alternative pathway in which L/F–tRNA–protein transferase (LFTR) adds a destabilizing N-terminal residue before the first methionine, degradation should probably not occur since the amino acids adjacent to methionine are alanine and glycine in PdT and His–TEV–PdT, respectively, and these two amino acids are not degradation prone (Dougan et al., 2010). Finally, all N-degron motifs reported by Humbard et al. (2013) were not found in both proteins. Accordingly, the ProtParam analysis at Expasy showed the estimated half-life in *E. coli* of both proteins to be >10 h, which is the maximum value. Therefore, we believe that it is very unlikely that degradation could be the reason for the differences observed.

Another interesting fact is that the His–TEV–PdT production was so intense that the plasmid was expulsed from the cells, which did not occur for PdT production. Despite the plasmid loss, His–TEV–PdT cultures returned the highest target protein concentrations, which should be related to plasmid loss during the culture on the first LB–Agar plate, which is performed in the absence of the antibiotic to allow all cells to grow. Thus, in this first plate without the antibiotic, the cells probably expulsed the plasmid due to the sudden removal of the selective pressure at the moment they face a strong metabolic burden related to heterologous protein synthesis. Therefore, no antibiotic-resistant cells were recovered on the second plate with the antibiotic (LB–Kan), returning a false negative result, as cell death or reduction in target protein production was not observed in the bioreactor, which certainly would have been observed if intense plasmid loss had occurred.

The *in silico* mRNA analyses suggested that the differences in target protein production were due to the mRNA structure at the 5′-end region. TIsigner revealed that the opening energy and expression score for PdT were 1.6 times higher and 2.7 times lower than those for His–TEV–PdT. The opening energy is inversely proportional to protein production levels (Bhandari et al., 2021b), and the His–TEV–PdT titer was 1.5-fold and 1.7-fold higher than for PdT, respectively, under C1 and C2 (Table 3), which is in agreement with the difference observed in the opening energy. Furthermore, RNAfold revealed that the MFE of the mRNA structure of the *pdt* gene version 1 was 2.2 times lower than that of the *his-tev-pdt* gene and a predicted secondary structure with a long stem that includes the *pdt* start codon, which also corroborated with the other results obtained. Altogether, this could indicate that during PdT production, the ribosome faced more difficulties during translation to unfold an mRNA secondary structure that is more stable than that of the *his-tev-pdt* mRNA, which certainly slowed down the protein production.

To investigate whether the cause of the observed difference in production was due to the initial region of the mRNA, we analyzed another gene construct (version 2) to produce PdT, with an identical amino acid sequence but a distinct 5′ mRNA region. The TIsigner results showed a 1.4-fold higher opening energy for the *pdt* gene version 1 compared to the *pdt* version 2, which is in accordance with the experimental results as the PdT production was 1.2-fold greater with the gene version 2 than with the gene version 1 (Table 5). Comparing the 5′ mRNA region of the *pdt* version 2 to the *his-tev-pdt*, a 1.1-fold higher opening energy was calculated. This result is also in accordance with the experimental data, which returned a 1.2-fold increase in target protein production for His–TEV–PdT when compared to PdT obtained with the gene version 2. In addition, the MFE calculated using RNAfold was −5.20 kcal/mol for the *pdt* version 2, which is between the values obtained for the *pdt* version 1 and *his-tev-pdt*, and the target protein production was also an intermediary value (Table 5). Finally, the MFE secondary structure predicted by RNAfold (Figure 6, left panel) shows a completely free start codon when compared to the other two sequences, which may have a great positive impact on protein production as, at the end of the third codon, an even longer stem structure is observed, which might have prevented reaching a better production with the *pdt* version 2. This observation indicates the relevance of having at least the two first codons free for ribosome binding. Moreover, although the *pdt* versions 1 and 2 have differences in the entire gene due to codon usage optimization of version 1, it was reported that changes in the first nine codons can achieve nearly optimum accessibility when compared to full-length modifications (Bhandari et al., 2021b). Therefore, codon differences in regions other than the 5′-end of the *pdt* version 2 are not likely to promote a significant reduction in opening energy and, consequently, should not increase protein production. It is also interesting to highlight that in this work, codon usage optimization worsened target protein production, showing that the mRNA initial region may play a much more important role in protein synthesis than generally supposed.

In this study, the difference between PdT and His–TEV–PdT was only the presence of the His-tag and TEV cleavage site sequences. As shown in Figure 5 (bottom panel), the ribonucleotides related to the His-tag sequence seemed to have a major impact on this result since only the last two codons of the TEV cleavage site were considered in RNAfold analysis. The region from −30 to +30 was considered because this region was employed to calculate the MFE in previous studies (Bhandari et al., 2021b). The prediction of the entire mRNA structure would generate a different structure that would be much complex to analyze since we do not know for how long the complete mRNA remains stable until degradation starts. It is also unknown how relevant this time interval for production is, compared with the translation that occurs in parallel with transcription. In addition, other studies have reported that the presence of the N-terminal His-tag improved the target protein synthesis, suggesting that the mRNA structures that contain the His-tag codons at the 5′-end region can be translated easier and faster than without these additional codons, increasing the amount of target protein obtained (Doray et al., 2001; Cèbe and Geiser, 2006; Wang et al., 2013; Park et al., 2015). Nonetheless, it is important to highlight that the phenomenon reported here was not provoked by the inclusion of the N-terminal His-tag itself. If the mRNA secondary structure without this tag has a higher MFE and lower opening energy, as a consequence, a more favorable translational structure is obtained, resulting in a higher expression score compared to the counterpart with the His-tag, as the His-tag insertion would worsen the target protein production.

The literature shows that TIsigner presented approximately 70% accuracy when predicting successes or failures of 11,430 expression experiments from a data bank and allowed four times higher production of the green fluorescent protein (GFP) and 1.5 times higher production of luciferase (Bhandari et al., 2021b). This tool also correctly predicted our experimental production differences, which supports that it should be widely used for optimization of recombinant protein production to save money and time in research and industry. We showed that instead of classical process optimization, which would take a much longer time, the titer can be increased optimizing the mRNA fold by changing the codons of the first amino acids. In comparison, the application of TIsigner software could provide optimized protein production in a much shorter time, possibly achieving even better results than those obtained using traditional process optimization strategies. Finally, it is important to highlight that this strategy is rarely applied neither to explain the results nor to improve production, and the case study presented here can contribute to disseminate this knowledge.

## Data Availability

The original contributions presented in the study are included in the article/Supplementary Material; further inquiries can be directed to the corresponding author.
