# Supplementary material for: Influence of the mRNA initial region on protein production: a case study using recombinant detoxified pneumolysin as a model
Source: Front Bioeng Biotechnol. 2024 Jan 8;11:1304965. doi: 10.3389/fbioe.2023.1304965 (PMC10800503; doi:10.3389/fbioe.2023.1304965)
Supplement: Supplementary file 1 [file DataSheet1.pdf]

Supplementary Material

Influence of the mRNA initial region on protein production: a case study using recombinant detoxified pneumolysin as a model

Filipe Fusco, Manuella Cazelato Pires, Alexandre Paulo Yague Lopes, Vítor dos Santos Alves and Viviane Maimoni Gonçalves*

*** Correspondence:** Viviane Maimoni Gonçalves: viviane.goncalves@butantan.gov.br

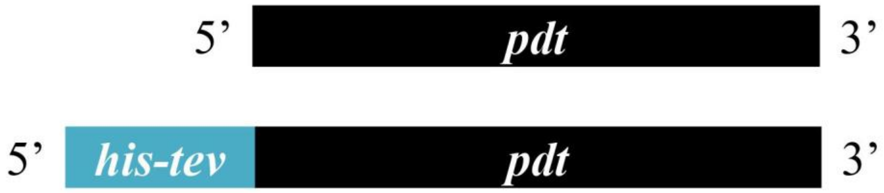


```
pdt   version 1   TTTTGTTTAACTTTAAGAAGGAGATATACCATGGCCAATAAAGCCGTGAACGATTTTAT  59
pdt   version 2   ...................................A........A..A..T..C.....  59
his-tev-pdt       ..................................T..CC.CCAT.A.C.T.ACGAAA.C  59
```

Supplementary Figure S1. Upper panel: Schemes of *his-tev-pdt* and *pdt* gene sequences. Bottom panel: Alignment of 5’end (-30 to +30) of *his-tev-pdt*, *pdt* version 1, and *pdt* version 2 gene sequences. Dots represent identical nucleotides.

Supplementary Table S1. Genetic sequences for *his-tev* and *pdt*. The *his-tev-pdt* start codon is in orange, His-tag codons in blue, and *Bam* H1 restriction site in red. To construct the *his-tev-pdt* sequence, the *his-tev* sequence was added to the *pdt* sequence without the first ATG codon (in green).

Name	Genetic sequence
his-tev	**ATG**GGTCACCACCATCATCATCACGAAAACCTGTATTTCCAGGGATCC
pdt	**ATG**GCCAATAAAGCCGTGAACGATTTTATTCTGGCCATGAACTACGAC<br>AAAAAAAAGCTGCTGACCCATCAGGGTGAAAGCATTGAAAATCGCTTT<br>ATCAAAGAAGGTAATCAGCTGCCGGATGAATTTGTTGTGATTGAACGT<br>AAAAAACGTAGCCTGAGCACCAATACCAGCGATATTAGCGTTACCGCA<br>ACCAATGATAGCCGTCTGTATCCGGGTGCACTGCTGGTTGTTGATGAAA<br>CCCTGCTGGAAAATAATCCGACACTGCTGGCAGTTGATCGTGCACCGAT<br>GACCTATAGCATTGATCTGCCTGGTCTGGCAAGCAGCGATAGCTTTCTG<br>CAGGTTGAAGATCCGAGCAATAGCAGCGTTCGTGGTGCAGTTAATGAT<br>CTGCTGGCAAAATGGCATCAGGATTATGGTCAGGTTAATAATGTTCCGG<br>CACGTATGCAGTATGAAAAAATCACCGCACATAGCATGGAACAGCTGA<br>AAGTTAAATTTGGCAGCGATTTTGAAAAAACCGGCAACAGCCTGGATA<br>TCGATTTCAATAGCGTTCATAGCGGTGAAAAGCAGATTCAGATTGTGAA<br>CTTCAAGCAGATCTATTACACCGTTAGCGTTGATGCAGTTAAAAATCCG<br>GGTGATGTGTTTCAGGATACCGTTACCGTGGAAGATCTGAAACAGCGT<br>GGTATTAGCGCAGAACGTCCGCTGGTGTATATTTCAAGCGTTGCATATG<br>GTCGTCAGGTGTATCTGAAACTGGAAACCACCAGTAAAAGTGATGAAG<br>TTGAAGCAGCATTTGAGGCCCTGATTAAAGGTGTTAAAGTTGCACCGCA<br>GACCGAATGGAAACAAATTCTGGATAACACCGAAGTGAAAGCGGTTAT<br>TCTTGGTGGTGATCCGAGTAGCGGTGCACGTGTTGTGACCGGTAAAGTT<br>GATATGGTTGAGGATCTGATTCAAGAAGGTAGCCGTTTTACCGCAGATC<br>ATCCGGGTCTGCCGATTAGCTATACCACCAGTTTTCTGCGTGATAATGT<br>TGTTGCAACCTTTCAGAATAGCACCGATTATGTTGAAACCAAAGTTACC<br>GCATATCGCAATGGCGATCTGCTGCTGGATCATAGTGGTGCATATGTTG<br>CACAGTATTATATCACCTGGGATGAGCTGAGCTATAACCATCAGGGCA<br>AAGAAGTTCTGACCCCGAAAGCATGGGATCGTAATGGTCAGGATCTGA<br>CCGCACATTTTACCACCTCAATTCCGCTGAAAGGTAATGTTCGTAATCT<br>GAGCGTTAAAATTCGTGAAGGCACCGGTCTGGCATTTGAATGGTGGCG<br>TACCGTTTATGAGAAAACCGATCTGCCTCTGGTTCGTAAACGTACCATT<br>AGCATTTGGGGTACAACCCTGTATCCTCAGGTGGAAGATAAAGTGGAA<br>AACGACTAA

Supplementary Table S2. Amino acid sequences for His-TEV-PdT and PdT. Blue: His-tag, pink: TEV protease recognition site, * stop codon.

Name	Amino acid sequence
His-TEV-PdT	MGHHHHHHENLYFQGSANKAVNDFILAMNYDKKKLLTHQGESIENRFIKEGNQLPDEFVVIERKKRSLSTNTSDISVTATNDSRLYPGALLVVDETLLENNPTLLAVDRAPMTYSIDLPGLASSDSFLQVEDPSNSSVRGAVNDLLAKWHQDYGQVNNVPARMQYEKITAHSMEQLKVKFGSDFEKTGNSLDIDFNSVHSGEKQIQIVNFKQIYYTVSVDAVKNPGDVFQDTVTVEDLKQRGISAERPLVYISSVAYGRQVYLKLETTSKSDEVEAAFEALIKGVKVAPQTEWKQILDNTEVKAVILGGDPSSGARVVTGKVDMVEDLIQEGSRFTADHPGLPISYTTSFLRDNVVATFQNSTDYVETKVTAYRNGDLLLDHSGAYVAQYYITWDELSYNHQGKEVLTPKAWDRNGQDLTAHFTTSIPLKGNVRNLSVKIREGTGLAFEWWRTVYEKTDLPLVRKRTISIWGTTLYPQVEDKVEND*
PdT	**M**ANKAVNDFILAMNYDKKKLLTHQGESIENRFIKEGNQLPDEFVVIERKKRSLSTNTSDISVTATNDSRLYPGALLVVDETLLENNPTLLAVDRAPMTYSIDLPGLASSDSFLQVEDPSNSSVRGAVNDLLAKWHQDYGQVNNVPARMQYEKITAHSMEQLKVKFGSDFEKTGNSLDIDFNSVHSGEKQIQIVNFKQIYYTVSVDAVKNPGDVFQDTVTVEDLKQRGISAERPLVYISSVAYGRQVYLKLETTSKSDEVEAAFEALIKGVKVAPQTEWKQILDNTEVKAVILGGDPSSGARVVTGKVDMVEDLIQEGSRFTADHPGLPISYTTSFLRDNVVATFQNSTDYVETKVTAYRNGDLLLDHSGAYVAQYYITWDELSYNHQGKEVLTPKAWDRNGQDLTAHFTTSIPLKGNVRNLSVKIREGTGLAFEWWRTVYEKTDLPLVRKRTISIWGTTLYPQVEDKVEND*
